# Amanitins in Wild Mushrooms: The Development of HPLC-UV-EC and HPLC-DAD-MS Methods for Food Safety Purposes

**DOI:** 10.3390/foods11233929

**Published:** 2022-12-05

**Authors:** Isabel Barbosa, Cátia Domingues, Rui M. Barbosa, Fernando Ramos

**Affiliations:** 1 Faculty of Pharmacy, Azinhaga de Santa Comba, University of Coimbra, 3000-548 Coimbra, Portugal; 2 Faculty of Medicine, Institute for Clinical and Biomedical Research (iCBR), University of Coimbra, 3000-548 Coimbra, Portugal; 3 REQUIMTE/LAQV, R. D. Manuel II, Apartado, 55142 Oporto, Portugal; 4 Center for Neuroscience and Cell Biology, University of Coimbra, Rua Larga, 3004-504 Coimbra, Portugal

**Keywords:** mushroom toxins, α-amanitin, β-amanitin, HPLC, mass spectrometry

## Abstract

Mushroom poisoning remains a serious food safety and health concern in some parts of the world due to its morbidity and mortality. Identification of mushroom toxins at an early stage of suspected intoxication is crucial for a rapid therapeutic decision. In this study, a new extraction method was developed to determine α- and β-amanitin in mushroom samples collected from central Portugal. High-performance liquid chromatography with in-line ultraviolet and electrochemical detection was implemented to improve the specificity of the method. The method was fully validated for linearity (0.5–20.0 µg·mL^−1^), sensitivity, recovery, and precision based on a matrix-matched calibration method. The limit of detection was 55 µg mL^−1^ (UV) and 62 µg mL^−1^ (EC) for α-amanitin and 64 µg mL^−1^ (UV) and 24 µg mL^−1^ (EC) for β–amanitin. Intra- and inter-day precision differences were less than 13%, and the recovery ratios ranged from 89% to 117%. The developed method was successfully applied to fourteen *Amanita* species (*A.* sp.) and compared with five edible mushroom samples after extraction with Oasis^®^ PRIME HLB cartridges without the conditioning and equilibration step. The results revealed that the *A. phalloides* mushrooms present the highest content of α- and β-amanitin, which is in line with the HPLC-DAD-MS. In sum, the developed analytical method could benefit food safety assessment and contribute to food-health security, as it is rapid, simple, sensitive, accurate, and selectively detects α- and β-amanitin in any mushroom samples.

## 1. Introduction

Mushrooms have long attracted a great deal of interest in many areas of the food and biopharmaceutical industries due to their well-known nutritional [1] and medicinal properties [2,3,4,5,6]. Remarkably, more recently, mushrooms have also been implemented in mycoremediation as “clean technologies” for different types of pollutants [7]. However, the vast number and differences in mushroom species make it challenging to distinguish between edible and poisonous mushrooms. In fact, mushroom poisoning remains a serious concern for food safety and human health, being commonly associated with acute toxicity episodes, morbidity, and mortality. Among the most toxic mushroom species is *Amanita phalloides,* which accounts for more than 90% of mushroom poisoning deaths [8], with many unreported cases [9].

The percentage of mushroom toxins in each sample depends on the geographic location, the growth conditions, the amount of toxin ingested, and the genetic profile [10].

**Figure 1:**
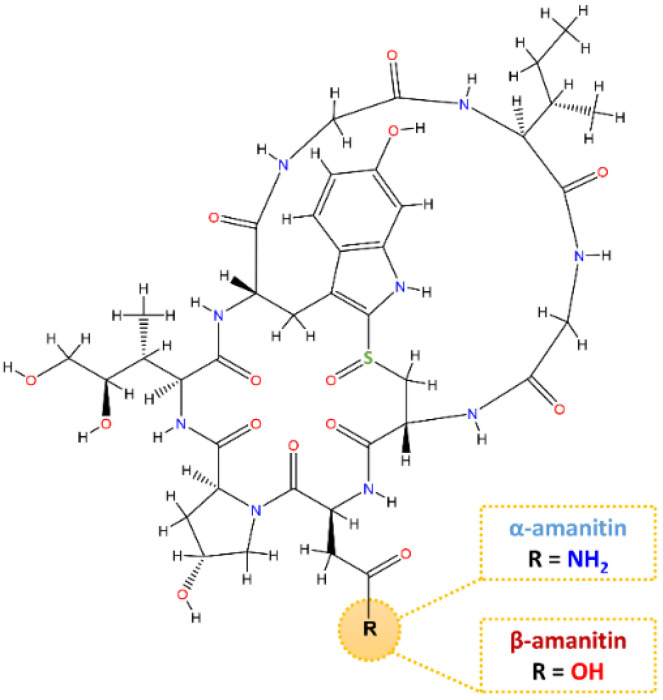
Chemical structure of α- and β-amanitin.

Mushroom toxins have been grouped into different categories, of which the cyclopeptides stand out [11]. The principal poisonous ingredient of the group of cyclopeptides is the amatoxins, namely the bicyclic octapeptides (Figure 1) from *Amanita* species [11,12], including α-and β-amanitin, which are 10–20 times more toxic than phallotoxins [13,14,15]. The structural formula of α-amanitin has been elucidated as a cyclic octapeptide whose ring is divided by a sulfoxide bridge from the original cysteine sulfur to the 2-position of the indole nucleus of a tryptophan unit (Figure 1). The three binding sites indispensable for the toxic effects of α-amanitin are (a) an isoleucine side chain in position 6, (b) a trans-4-hydroxyl group at proline in position 2, and (c) a hydroxylated L-isoleucine side chain in position 3 [16]. Besides α-amanitin, a carboxamide, β-amanitin has also been identified and isolated [16,17,18].

The underlying toxicological mechanisms are usually associated with RNA polymerase II inhibition, which may induce changes in normal cell metabolism, cell death, and tissue necrosis [19]. Despite recognizing the possible toxic pathways, discovery is a demanding process, making urgent the development of innovative therapies [20,21,22].

Moreover, considering the relevant number of publications using different methods to determine amatoxins in various mushroom and biological matrices, a gap in the food chain assessment of mushroom poisoning still persists.

Several studies have been described for the determination of amatoxins in wild mushrooms based on reverse-phase high-performance liquid chromatography (RP-HPLC) with different detection techniques, such as ultraviolet (UV) [23], diode array detection (DAD) [24,25,26], mass spectrometry MS-TOF [27], and DAD-MS [28,29,30]. Electrochemical detection (EC), either amperometric or coulometric, can be very attractive due to its specificity and sensitivity for the detection of easily oxidizable compounds in food, environmental, and biological samples [31,32,33]. Furthermore, the EC detectors are inexpensive, easy to assemble, and of high analytical robustness [31].

However, to the best of our knowledge, there is no reporting the assessment of toxin content in *Amanita* species collected from the central region of Portugal.

Therefore, the aims of this work were: (1) to develop a simple and quick analytical method for simultaneous detection of α- and β-amanitin by HPLC with in-line UV and electrochemical (EC) detection in order to improve the specificity of the method and (2) to apply the developed method in the detection of α- and β-amanitin in wild mushroom samples collected in the central region of Portugal. For that purpose, an (3) environmentally friendly solid-phase extraction (SPE-Oasis^®^ PIME HLB) method with high efficiency and selectivity was developed and optimized. Moreover, (4) to confirm the results obtained using the developed method, the HPLC-DAD-MS method was applied.

## 2. Materials and Methods

### 2.1. Reagents and Materials

All reagents and solvents were of analytical grade unless specified. The standards of α-amanitin (≥90% purity), β-amanitin (≈90% purity), and sodium acetate were acquired from Sigma–Aldrich (Steinheim, Germany). HPLC-grade methanol and acetonitrile were obtained from Merck (Darmstadt, Germany), and ultrapure water was supplied by a Milli-Q water purification apparatus (Millipore Lda, Bedford, MA, USA). All solutions prepared for HPLC were filtered using a 0.45 mm nylon filter. The Oasis^®^ PRIME HLB (1 mL/30 mg) from Waters Corp, (Mildford, MA, USA) and a vacuum manifold system (VaElut 6 Manifold Processing Station, Agilent Technologies, Santa Clara, CA, USA) were used.

### 2.2. Working Solutions, Calibration Curve, and Quality Control Samples

The stock solutions of α- and β-amanitin at the concentration of 1.0 mg mL^−1^ were prepared by diluting each standard in methanol. Standard solutions were diluted with 1 mL of mobile phase [0.1 mol/L sodium acetate, pH 4.7 (A): methanol (B), 83:17, *v*/*v*)] at the following concentrations for each amanitin: 0.5, 1.0, 2.0, 5.0, 10.0, and 20.0 μg mL^−1^. All stock solutions were stored at −20 °C in the dark until use. Quality Control (QC) samples were prepared at 0.5 μg mL^−1^ (low-quality control, LQC), 2.0 μg mL^−1^ (medium-quality control, MQC), and 10.0 μg mL^−1^ (high-quality control, HQC) for α- and β-amanitin, respectively. Quality control samples were prepared by spiking blank shiitake mushroom samples and treated according to the sample preparation described below.

### 2.3. Mushroom Collection

**Figure 2:**
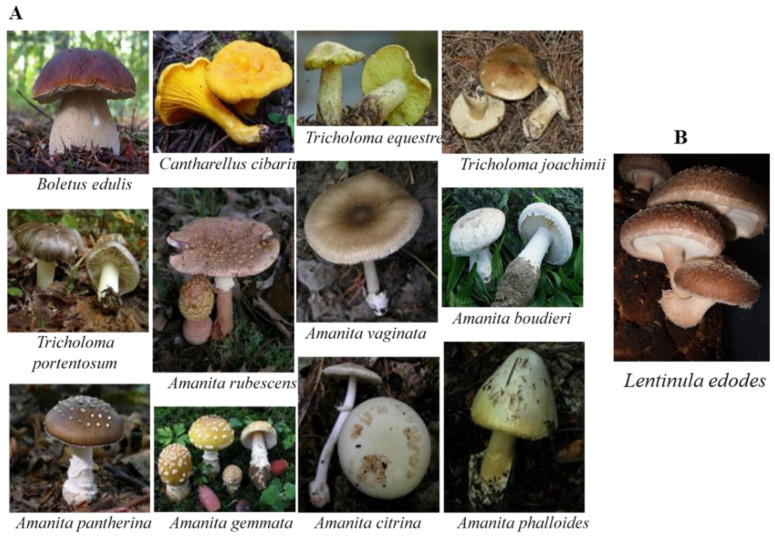
Pictures of (**A**) some wild mushrooms collected in the Inner Central Region of Portugal and (**B**) *Lentinula edodes* mushroom used in this work.

Mushroom samples were used to develop and validate the analytical method. For this, fourteen wild mushrooms of different phenotypic species were collected from green-open spaces in the Inner Center Region of Portugal (Figure 2) during September and November to be used as testing samples. Shiitake mushroom samples (Lentinula edodes) were purchased from local markets to serve as blank samples. After properly collecting the mushroom samples, they were adequately scrubbed, dried at 40 °C for 24 h, and stored at −20 °C to avoid degradation.

### 2.4. Sample Preparation and Extraction Procedure

**Figure 3:**
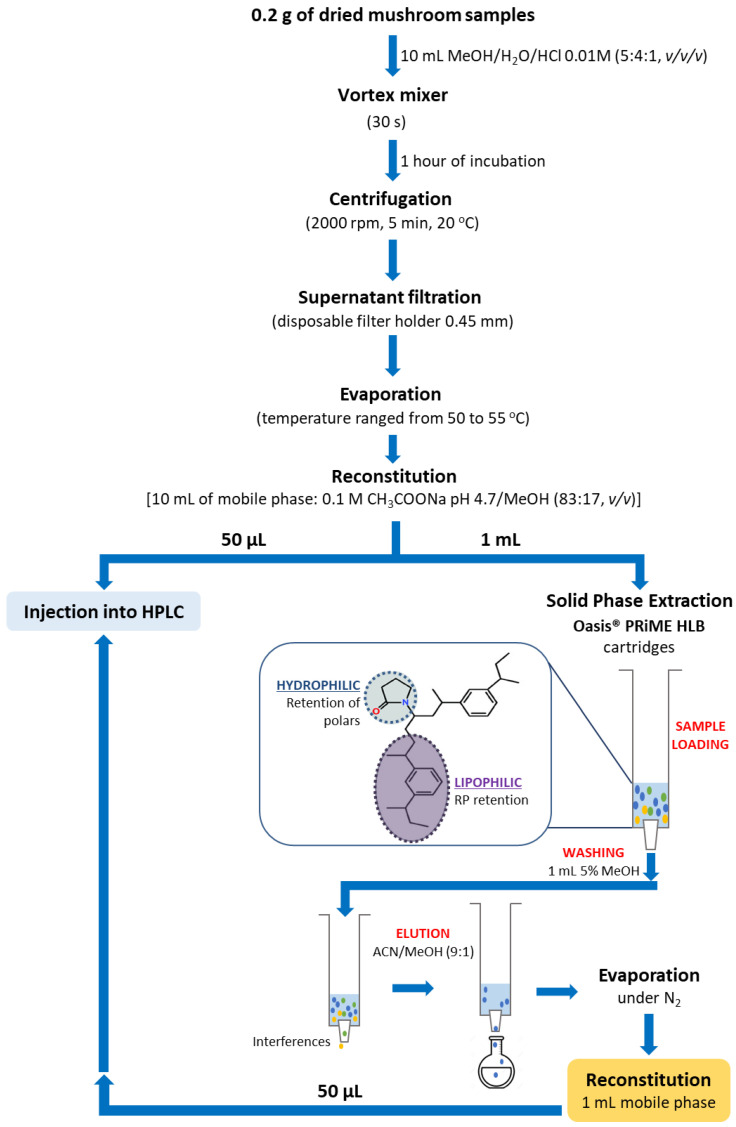
Flow chart showing the proposed sample treatment and analysis for HPLC-UV-EC.

A 0.2 g sample of all parts of the dried mushrooms was weighed, finely chopped, and placed in a test tube with 10 mL of extraction solvent [methanol/water/0.01 mol/L HCl (5:4:1, *v*/*v*/*v*)]. The mixture was vortexed for 30 s, incubated for 1 h at room temperature, and centrifuged at 2000× *g* for 5 min. The supernatant was collected, filtered by a disposable filter holder of 0.45 µm, and evaporated to dryness at 50–55 °C. The residue was reconstituted in 10 mL of mobile phase [83 (A):17 (B)]. The solid-phase extraction (SPE) of α- and β-amanitin was performed using Oasis^®^ PRIME HLB cartridges without conditioning the column or the equilibration step, as shown in Figure 3. One mL of the previously obtained solution was loaded onto an Oasis^®^ PRIME HLB extraction cartridge without preconditioning of the cartridge sorbent. Afterwards, the cartridge was washed with 1 mL of 5% methanol, and the elution was performed with 1 mL of acetonitrile: methanol (9:1). The eluate was collected and thoroughly dried to a solid residue under a slight nitrogen flow at room temperature. The residue was reconstituted with 1 mL of mobile phase, and 50 µL was injected into the chromatographic system. Blank shiitake mushroom samples alone and spiked (QC) were prepared in triplicate, and the SPE was then performed according to the scheme described in Figure 3.

### 2.5. Chromatographic System and Conditions

Quantification of amatoxins by HPLC with in-line UV and EC detection was performed using Flexar Perkin Elmer HPLC (Norwalk, CT, USA) chromatography comprising an LC Flexar binary pump, LC Flexar autosampler, and LC Flexar detector UV/VIS, followed by an electrochemical detector BASi (West Lafayette, IN, USA), coupled to a Perkin Elmer TotalChrom workstation. The chromatographic separation was done using a reverse-phase Brisa LC2 C18 column (150 mm × 2.1 mm, 3 µm particle size) at room temperature. The mobile phase was composed of a buffer mixture of 0.1 mol/L sodium acetate (pH 4.7) (A) and methanol (B) in a ratio of 83 (A):17 (B), *v*/*v*. Then, it was filtered through a 0.45 µm membrane (Schleicher & Schuell) and degassed. Isocratic elution was applied at a flow rate of 1.0 mL min^−1^, and the injection volume was 50 μL for all samples. A sequential detection was performed for all studies using UV detection at 305 nm, followed by an EC detection holding potential (+0.600 V vs. Ag/AgCl).

Identification of amatoxins by was performed by high-performance liquid chromatography, diode array, and mass spectrometry detection. The analysis of α-amanitin and β-amanitin was achieved using a Quadrupole/Ion Trap Mass Spectrometer (QIT-MS) (LCQ Advantage MAX, THERMO) coupled to a Liquid Chromatograph of High Performance (Finnigan Surveyor, THERMO). Separation was performed on a Spherisorb^®^ ODS-2 C18 column (150 mm × 2.1 mm; particle size 3 μm; Waters^®^ Corporation, Milford, MA, USA) and a Spherisorb^®^ ODS-2 guard cartridge C18 (10 mm × 4.60 mm; particle size 5 μm; Waters^®^ Corporation, Milford, MA, USA) at 25 °C, using 0.1 mol/L sodium acetate with a pH of 4.7 (eluent A) and methanol (eluent B) as a mobile phase. The gradient profile was 90% A/10% B (0.0–4.0 min), 82% A/18% B (4.0–10.0 min), 0% A/100% B (10.0–30.0 min), and 90% A/10% B (30–40 min), at a flow rate of 200 μL mL^−1^.

The DAD detector was used in a wavelength ranging from 200 to 600 nm and recorded at 305 nm. The mass detector was operated in the positive electrospray ionization (ESI) mode using selected reaction monitoring (SRM) acquisition. The source voltage was 5.00 kV, and the capillary temperature and voltage were 150 °C and 42 V, respectively. Nitrogen was used as nebulizing gas, with a sheath gas flow of 15 (arbitrary unit) and an auxiliary sweeps gas flow of 5 (arbitrary unit). The collision gas was helium, with a normalized collision energy of 35%. A precursor ion (MS1) and an MS2 product ion were obtained for α-amanitin and β-amanitin.

### 2.6. Validation of the Method

The method was validated according to the established and approved guidelines [34,35,36]. For that, selectivity, linearity, the limit of detection (LOD) and limit of quantification (LOQ), precision (intra- and inter-day repeatability), recovery, and matrix effect employing a matrix-matched calibration method were assessed.

#### 2.6.1. Selectivity

The selectivity was evaluated by comparing, under optimized chromatographic conditions, chromatograms of standard α- and β-amanitin, an extract of blank mushroom samples, and an extract of blank mushroom samples spiked with both amanitins to exclude interferences that could co-elute with amanitins. The absence of detectable interfering peaks at the α- and β-amanitin retention times was considered a lack of interference.

#### 2.6.2. Linearity

The linearity response for α- and β-amanitin was obtained by an external calibration using six standards in the mobile phase, injected three times, covering a range of 0.5–20.0 μg mL^−1^. Linear regression equations evaluated linearity for each curve. Correlation coefficients (R^2^) were determined, and residual analysis showed the straight-line model is correct [34,35,36].

#### 2.6.3. Precision

The intra-day precision was estimated by replicating analysis (n = 6) of QC samples at three concentration levels: 0.5, 2.0, and 10.0 μg mL^−1^. The inter-day precision (n = 3) was assessed by testing the same QC samples over three consecutive days. The coefficient of variation (CV) was calculated to estimate precision according to the following equation: *CV* (%) = (*σ*/*μ*) × 100, where σ is the standard deviation and μ is the mean of the response.

#### 2.6.4. Limit of Detection (LOD) and Limit of Quantification (LOQ)

According to the standard guidelines for analytical procedures, the LOD is the lowest amount of analyte in a sample that can be detected but not necessarily quantified as an exact value. The LOQ corresponds to the lowest amount of analyte in a sample which can be quantitatively determined with suitable precision and accuracy [34,35,36]. The LOD and LOQ for α- and β-amanitin by the proposed method were determined using fortified shitake mushroom samples, i.e., the matrix-matched calibration method (MMCM), and calculated using the standard deviation of the residuals (S_y/x_) of the regression line and the slope of the calibration curve (*m*) by the following equations: LOD = 3.3(S_y/x_/*m*) and LOQ = 10(S_y/x_/*m*).

#### 2.6.5. Recovery and Matrix Effect

The recovery and matrix effect were evaluated at three different concentrations: 0.5, 2.0, and 10.0 μg mL^−1^. The recovery (n = 6) was determined by comparing the response of extracted analyte in the spiked blank matrix with the response of standard solutions.

The matrix effect (n = 5) was evaluated by comparing the calibration graphs by spiking shiitake mushroom samples with known amounts of α- and β-amanitin and the calibration graph obtained from α- and β-amanitin standard solutions at the same concentration by the following equation: matrix effect (%) = (*m_QC_*/*m_ST_*) × 100, where *m_QC_* is the slope of fortified shiitake mushroom samples, and *m_ST_* is the slope of standard solutions of α- and β-amanitin.

### 2.7. Method Application

The validated method was applied to fourteen species of mushrooms collected in the Central region of Portugal. According to the method, all samples were pre-treated, extracted by SPE-Oasis^®^ PRIME HLB, and 50 µL of the residue was injected into HPLC-UV-EC to identify and quantify the presence of these toxins (Figure 3). The identity of the α- and β-amanitin was confirmed by comparing the retention times (t_R_), UV λmax, and MS^n^ data with those of known standard references by HPLC-DAD-MS.

## 3. Results and Discussion

### 3.1. Optimization of Electrochemical Detection

The presence of a 6-hydroxytryptophan, an indole nucleus, in α- and β-amanitins makes it possible to detect these compounds by using an electrochemical detector operating in an oxidative mode [37,38]. EC detectors are usually more sensitive and selective than a standard UV detector, although they disadvantageously require more time to stabilize [31,32].

**Figure 4:**
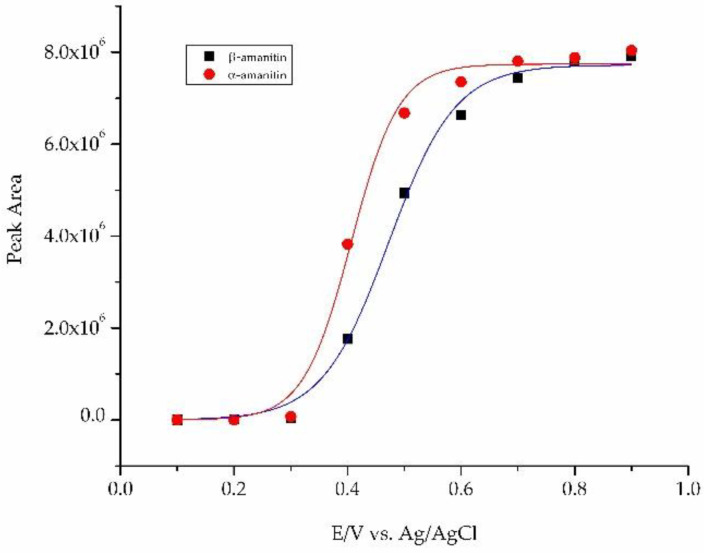
Hydrodynamic voltammograms of α- and β-amanitin standard solutions. Peak areas are plotted versus electrode potentials.

Electrochemical studies were conducted using standard solutions of both toxins and a wall-jet cell detector comprising a glassy carbon working electrode with potentials ranging between 0.100 to 1.00 V using Ag/AgCl as the reference electrode. This experimental design made possible the establishment of the most suitable electrode-holding potential in order to reduce the background current and improve the sensitivity and selectivity of the method. Figure 4 depicts the hydrodynamic voltammograms obtained by plotting the peak area versus the working electrode potential.

The half-wave potentials calculated by fitting data to a sigmoidal function for each curve were very similar for α-amanitin (+0.406 V) and β-amanitin (+0.472 V), which are slightly lower values (ca. 50 mV) compared to those previously achieved [39]. The potential of +0.600 V provides an optimal compromise between sensitivity and selectivity in detecting both amanitins, namely α-amanitin. However, it may not compromise the selectivity and sensitivity of the method, as it has been reported that the use of electrode potentials of +350 mV presents enough sensitivity and selectivity to detect α-amanitin in human plasma samples [38]. In another study, coulometric detection using working electrodes with high surface areas at an oxidation potential of +0.500 V has also been used to quantify α-amanitin in urine [37] and biological samples such as liver and kidney [13]. Interestingly, to circumvent issues regarding EC sensitivity, it has been recommended to regularly polish the electrode surface, as products derived from the indole residue of amatoxins can accumulate there, compromising the results.

### 3.2. Optimization of Sample Extraction Procedures

The extraction of the analytes of interest from a biological matrix or a mushroom sample is critical in developing an analytical method because it will affect the overall sensitivity, selectivity, and accuracy [40].

Several procedures for the extraction of toxins from mushroom samples have been reported. α- and β-amanitin and phalloidin have been extracted under acidic conditions, such as 0.1% trifluoroacetic acid in methanol [30], 0.5% acetic acid in methanol [41], and 2.5% formic acid in acetonitrile [42], methanol [43], methanol/water (MeOH/H_2_O; 1:1) [44,45], and acidified methanol/water (MeOH/H_2_O/(HCl or HCOOH) 0.01 M; 5:4:1) by the addition of mineral or organic acid [23,46,47]. Thus, extraction solvents reported in the literature are different according to the mushroom toxins [27].

In the present work, the mushroom sample preparation was performed in two steps before the injection into the HPLC system. First, the mushroom samples were crushed and homogenized with acidified aqueous methanolic solutions. Then, the extracted solution was cleaned up by one-step PRIME (process, robustness, improvements, matrix effects, ease of use). The Oasis^®^ PRIME HLB cartridge is a proprietary sorbent based on Oasis^®^ HLB technology that does not require any solvation, equilibration, or conditioning step, thus saving time and solvent expenses, being that β-amanitin, an acidic compound (-OH group on aspartic residue), elutes earlier than α-amanitin, a neutral compound (-NH_2_ group on aspartic residue). The recovery rates and sensitivity were higher using methanol. The effective PRIME pass-through clean-up procedure using the Oasis^®^ PRIME HLB cartridge was optimized. The results demonstrated higher extraction capacity with recoveries in 80 to 100% (n = 5). Literature is scarce regarding the use of SPE cartridges for extraction and purification of α- and β-amanitin in mushroom samples. Sample purification procedures using Oasis HLB [27,30], Oasis^®^ MAX [27,30,42], Oasis^®^ WAX [27,30,41], and Sep-Pak Plus Accell QMA [30] have been described. Oasis^®^ HLB gave the highest recovery of Amanita toxins extracted from mushrooms. Oasis^®^ PRIME HLB has been described for extraction and purification of these toxins in biological samples [48].

Our results agree with those reported by Zhang et al. using biological samples (plasma, serum, and urine) for α-amanitin, β-amanitin, γ-amanitin, phalloidin, and phallacidin [45]. The optimized pre-treatment procedure using the Oasis^®^ PRIME HLB method provides great recoveries and reduced matrix interference. This procedure is favorable, as it is considered environmentally friendly when compared with other SPE cartridges or other extraction procedures [49].

### 3.3. Optimization of the Chromatographic Conditions

Chromatographic separation was carried out with a reverse-phase column Brisa LC2 C18 (150 mm × 2.1 mm, 3 µm) under isocratic conditions. Other parameters, including the composition and the ratio of mobile phase, flow rate, and column temperature, were also optimized to obtain a symmetric peak shape and maximum intensity. The hydrophobic stationary phase retains lipophilic analytes and separates a wide range of compounds (charged/non-charged and polar/apolar). Moreover, it offers an excellent lifetime under extreme pH conditions (including pH 4.7) and allows a rapid elution of compounds. Absorption spectra of α- and β-amanitin showed a maximum absorption peak at 305 nm due to the 6-hydroxyTrp [23,50]. Phosphate or citrate buffers have been used as mobile phase, even though precipitation can occur and may compromise the HPLC valves [13,51]. Therefore, we used acetate buffer (pH 4.7):methanol (83:17, *v*/*v*) at a flow rate of 1 mL min^−1^ in an overall analysis time of 25 min.

The chromatographic conditions used for HPLC-DAD-MS were also optimized to obtain an efficient separation and identification of amanitins in wild mushroom samples. In addition, several MS parameters have been optimized for greater sensitivity. HPLC-DAD-MS analyses were performed to identify the main amatoxins (α- and β-amanitin) present in the wild mushroom extracts.

### 3.4. Validation of the Method

Under optimized conditions, the retention time of β- and α–amanitin were ca. 16 min and 19 min, respectively. Blank shiitake mushroom samples, alone and spiked with α- and β-amanitin, presented no interfering peaks at the α- and β-amanitin retention times.

The calibration curves obtained using standard solutions with six concentrations of each amanitin were linear over the range of 0.5 to 20.0 µg mL^−1^, with appropriate correlation coefficients (Table 1).

The estimated LOD and LOQ values of HPLC-UV-EC detection for α- and β-amanitins are shown in Table 2. As expected, the LOD and LOQ for β-amanitin are lower for EC detection compared to UV. However, no significant differences were observed for α-amanitin for either EC or UV. Therefore, electrochemical detection complements the method, improving selectivity compared to UV detection at 305 nm for both amanitins. According to the literature, the analytical methods used for the analysis of α- and β-amanitin, especially α-amanitin, based on HPLC with electrochemical detection, are scarce and have been performed in biological samples [37,38,39]. Therefore, comparing the obtained LOD and LOQ values with those reported in the literature is challenging, because in most cases, methodological details are poorly described or omitted. Table 2 aims to review the LOD obtained from the literature for α- and β-amanitin in mushroom extracts. As can be observed HPLC-DAD-MS showed LODs in the range of 2–230 ng g^−1^, based on a signal-to-noise ratio of 3. It should be noted that the LOD and LOQ obtained in the present work were calculated based on the calibration curve obtained from the MMCM and produced more conservative estimations.

Table 3 summarizes the overall recovery and the matrix effect of HPLC-UV-EC assay of α- and β-amanitin for the shiitake mushroom extracts, expressed as the percentage of recovery and relative standard deviation (%RSD). As shown, recoveries of α- and β-amanitin ranged from 89% to 117%, with an RSD < 5%. The method presents acceptable extraction efficiency with reproducible recovery since the losses of both amanitins were minimal during the extraction procedure, even for the lowest concentration.

The matrix effect was evaluated by comparing the slopes of calibration curves obtained when processing spiked shiitake mushroom samples and standard solutions of α- and β-amanitin at the same concentrations. The results obtained were in the range of 95.8% to 97.6% for low, medium, and high concentrations of α- and β-amanitin, respectively (Table 3), with an RSD < 9% for α-amanitin and an RSD < 12% for β-amanitin.

The intra-day and inter-day precision for α-amanitin and β-amanitin in mushroom matrices ranged from 0.41% to 12.95% (Table 4), within the requested limit (%RSD < 20%).

The results summarized in Table 3 and Table 4 indicated that this method showed good precision and accuracy for α- and β-amanitin obtained from mushroom samples.

### 3.5. Method Application

After developing and validating the HPLC-UV-EC method, it was applied for quantifying α- and β-amanitin in different wild mushroom samples collected in the central region of Portugal. The results showed the presence of α- and β-amanitin in the samples of *A. phalloides*. No α- or β-amanitin were detected in any other species analyzed, which has been confirmed by HPLC-DAD-MS. The concentration of α- and β-amanitin in the extract of *A. phalloides* is shown in Table 5 and is similar for EC and UV detection modes. Our results are consistent with previous report of α- and β-amanitin content of *A. phalloides* analyzed by HPLC-UV [23]. Garcia et al. reported the composition and distribution of amatoxins and phallotoxins in different tissues of *A. phalloides* from two other locations in Portugal using LC-DAD-MS [29]. The content and composition of cyclopeptides in lethal amanitins have been shown to vary between different species [45]. Previous studies have also shown that climate, topography, soil characteristics, body tissue, and growth stages influence the concentrations and distributions of amatoxins and phallotoxins in *A. phalloides* [24,46].

Under the optimized qualitative conditions, all mushroom extracts were analyzed in positive ion mode using HPLC-DAD-MS to identify and confirm the presence of α- and β-amanitin. The chemical structures of the α- and β-amanitin are illustrated in Figure 1. The identification of α- and β-amanitin peaks under UV was based on their retention times, spectra, molecular ion characteristics, and fragment ions. Under these conditions, the base peaks of the mass spectra were *m*/*z* 919.13 (C_39_H_54_N_10_O_14_S) for α-amanitin and *m*/*z* 920.37 (C_39_H_53_N_9_O_15_S) for β-amanitin (Table 6).

**Figure 5:**
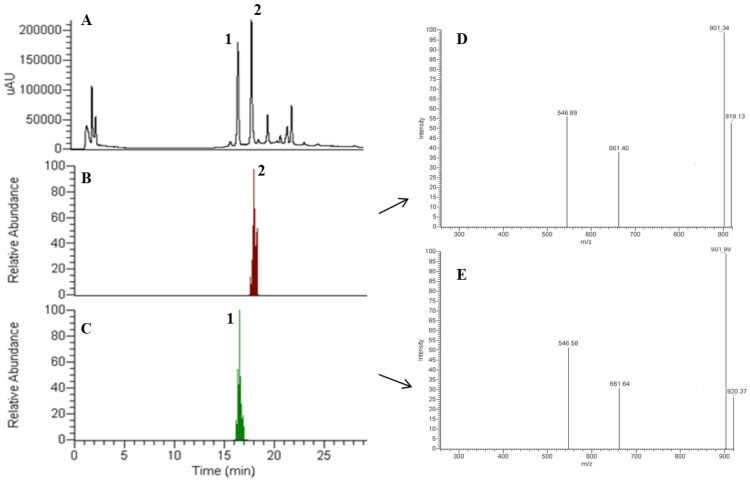
(**A**) HPLC-DAD chromatogram of an extract of Amanita phalloides, monitored at 305 nm, with the separation of peaks of β-amanitin (1) and α-amanitin (2). Total ion chromatogram (TIC) (**B**) and mass spectrum and fragmentation pattern (**D**) of peak 2-α-amanitin and total ion chromatogram (TIC) (**C**) and mass spectrum and fragmentation pattern (**E**) of peak 1-β-amanitin with a retention time of 18.1 and 16.6 min, respectively.

The chromatogram obtained by HPLC-DAD of the extract of Amanita phalloides confirmed the presence of α- and β-amanitin (Figure 5A). Both compounds exhibited an absorption maximum at 303–305 nm, similar to α- and β-amanitin patterns [23]. As the backbone of α-amanitin and β-amanitin is a bicyclic octapeptide, and since the only structural difference between α- and β-amanitin resides in the -NH_2_ for α-amanitin and -OH for β-amanitin groups, it is reasonable to expect that both compounds undergo a similar fragmentation pathway under optimized MS conditions. Therefore, we have studied and compared the fragment ions of α- and β-amanitin.

Based on the chromatographic peaks obtained by DAD and total ion chromatogram (TIC), the molecular ion [M+H]^+^ at *m*/*z* 919 (relative intensity, 100%) corresponds to the protonated molecular ion of α-amanitin (Figure 5C). The [M+H]^+^ ion was further selected and isolated to pass through the collision cell to produce MS^2^ fragment ions (Figure 5E), and the results showed only three fragment ions (Table 6). The limited fragmentation of α- and β-amanitin by the positive ionization mode agrees with a previous work of Johnson et al. [50,53]. The MS^1^ and MS^2^ mass spectra of β-amanitin were acquired under the same conditions, and [M+H]^+^ ions were observed at *m*/*z* 920 (Figure 5B,D).

**Figure 6:**
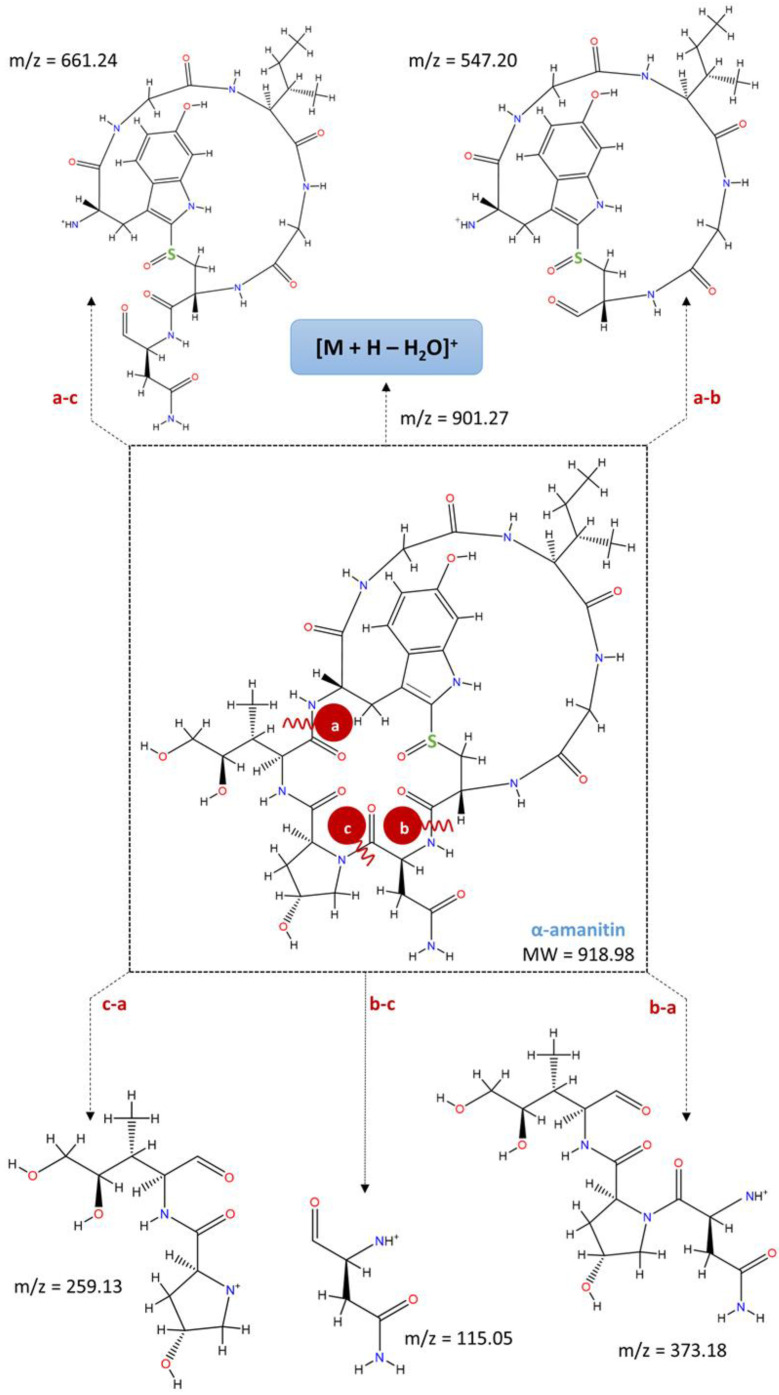
Schematic representation of the fragmentation sites in the chemical structure of α-amanitin and the respective characteristic fragments produced with the corresponding *m*/*z* values.

A comparison of the MS^2^ spectra fragmented ions revealed some differences, summarized in Table 6. The α- and β-amanitin mass spectra exhibited characteristic fragments at *m*/*z* 901 and 902, respectively, produced by dehydration of protonated ions [M + H-H_2_O]^+^. However, it is not well-known which hydroxyl group is lost from the parent structure when collision-induced dissociation occurs. These fragmented ions agree with previous studies [30,42,51,54,55] and can be used as characteristic ions to identify α- and β-amanitin in complex sample matrices. MS^2^ data also showed ion fragments at *m*/*z* 661 [fragment a to c + H]^+^ and *m*/*z* 547 [fragment a to b + H]^+^, typical of α- and β-amanitin, as shown in Figure 6. Since the same ions fragments were produced with identical mass differences, it is predictable that α- and β-amanitin have a similar cleavage pathway. Figure 6 shows a schematic representation of fragmentation sites in the chemical structure of α-amanitin and the respective characteristic fragments produced [30,51]. Cleavage of peptide bonds at dihydroxy-Ile-Gly (a), Asn-Cys (b), and hydroxyl-Pro-Asn (c) also have the ion peaks at *m*/*z* 373 [fragment b to a + H]^+^, 259 [fragment c to a + H]^+^, and 115 [fragment b to c + H]^+^, which can be observed in spectra of the amanitin fragmentation mass. The MS/MS mode for data acquisition provided a high specificity and allowed an unequivocal detection and confirmation of amanitins in wild mushroom samples, namely in *A. phalloides*.

## 4. Conclusions

Developing a simple and accurate analytical method for determining amatoxin content in wild mushrooms is of interest for treating cases involving poisoning. In this work, a simple, sensitive, accurate, rapid, and affordable method was developed for the simultaneous determination of α- and β-amanitin in wild mushrooms collected in the center of Portugal. The method based on HPLC-UV-EC involved the simultaneous extraction of both toxins using Oasis^®^ PRIME HLB sorbent, selected for clean-up due to its simplicity, cost-effectiveness, and adequate recoveries. The method was validated and successfully applied for analyses of fourteen wild mushrooms samples. High levels of α- and β-amanitin were detected in *A. phalloides* samples, which was confirmed by HPLC-DAD-MS. The HPLC-UV-EC method offers several advantages: (1) its applicability in routine analysis to detect and quantify toxins in wild mushrooms with sensitivity and specificity; (2) a rapid identification of α- and β-amanitin in amatoxin-containing species poisoning, thus contributing to a faster application of the adequate treatment, and (3) the identification of these toxins in wild mushroom samples constitutes an up-to-date step to increased food safety and reduced risk to human health.

## Figures and Tables

**Table 1:** Calibration curve parameters of α- and β-amanitin; regression equation and coefficient (R^2^) are expressed as the mean of 4 calibration curves (n = 4).

Compound	Detection	Range (µg mL^−1^)	Linearity (R^2^)	Linear Regression Equation
α-amanitin	EC	0.5–20.0	0.9998	y = 621,020x + 10,701
	UV	0.5–20.0	0.9993	y = 36,624x + 5896.6
β-amanitin	EC	0.5–20.0	0.9997	y = 346,265x + 31,251
	UV	0.5–20.0	0.9996	y = 27,942x + 1373.6

Equation of the calibration curve is given by the general equation of y = mx + b, with y corresponding to the peak area, m to the slope, and b to the intercept.

**Table 2:** Limits of detection for direct mushroom extracts.

Matrix	Mushroom Toxin	LOD (ng g^−1^)	LOQ (ng g^−1^)	Method	Reference
Mushroom tissue	α-amanitin β-amanitin	EC-62 UV-55 EC-24 UV-64	EC-189 UV-168 EC-72.6 UV-193	HPLC-UV-EC	Present Work
Mushroom tissue	α-amanitin β-amanitin	20 20	NS	HPLC-ESI-MS	[42]
Mushroom tissue	α-amanitin β-amanitin	30 30	NS	HPLC-TOF-MS	[30]
Mushroom tissue	α-amanitin β-amanitin	230 190	26.8 33.3	HPLC-TOF-MS	[27]
Mushroom tissue	α-amanitin β-amanitin	2 2	NS	HPLC-TOF-MS	[52]

NS: not specified.

**Table 3:** Recovery and matrix effect values for α- and β-amanitin analyzed by HPLC-UV-EC.

Compound	Detection	Concentration (µg mL^−1^)	Recovery (%) (RSD)	Matrix Effect (%) (RSD)
α-amanitin	EC	0.5	117 (4.78)	96.9 (8.01)
		2.0	99.4 (1.37)	
		10.0	96.1 (1.48)	
	UV	0.5	104 (3.72)	95.8 (8.87)
		2.0	92.5 (1.64)	
		10.0	96.8 (0.34)	
β-amanitin	EC	0.5	114 (3.25)	95.8 (11.4)
		2.0	103 (2.94)	
		10.0	93.6 (0.48)	
	UV	0.5	92.0 (3.9)	97.6 (10.9)
		2.0	89.0 (2.7)	
		10.0	93.3 (0.6)	

**Table 4:** Intra- and inter-day precision for values for α- and β-amanitin analyzed by HPLC-UV-EC.

Compound	Detection	Concentration (μg mL^−1^)	Precision (%RSD)	
			Intra-day	Inter-day
α-amanitin	EC	0.5	5.35	12.95
		2.0	1.05	7.64
		10.0	0.41	9.95
	UV	0.5	10.23	3.73
		2.0	1.13	2.46
		10.0	0.62	11.75
β-amanitin	EC	0.5	4.20	10.38
		2.0	3.34	5.45
		10.0	1.22	3.81
	UV	0.5	5.33	5.80
		2.0	4.55	3.69
		10.0	0.69	3.51

**Table 5:** Amanitin identification and quantification in Portuguese wild mushrooms.

Mushroom Species	HPLC-UV-EC
*Amanita citrine;*	
*Amanita pantherine;*	
*Amanita boudieri;*	
*Amanita gemmate;*	
*Amanita muscaria;*	
*Amanita rubescens;*	
*Amanita vaginatae;*	ND
*Amanita var alba;*	
*Boletus edulis;*	
*Cantharellus cibarius;*	
*Tricholoma equestre;*	
*Tricholoma joachimii;*	
*Tricholoma portentosum*	
*Amanita phalloides*	α-amanitin: (EC) < > 290 µg g^−1^ β-amanitin: (EC) < > 280 µg g^−1^ α-amanitin: (UV) < > 280 µg g^−1^ β-amanitin: (UV) < > 288 µg g^−1^

ND: Not detected.

**Table 6:** Retention time and fragment ions of α- and β-amanitin.

Compounds (Formula)	Molecular Weight	Retention Time (min)	Protonated Molecular Ions (MS^1^, *m*/*z*)	Major Fragment Ions (MS^2^, *m*/*z*)
α-amanitin (C_39_H_54_N_10_O_14_S)	918.97	18.1	[M+H]^+^ 919.00	901.14 (100%) 661.43 (44.8%) 546.71 (60%)
β-amanitin (C_39_H_53_N_9_O_15_S)	919.95	16.6	[M+H]^+^ 920.35	902.09 (100%) 661.77 (45%) 546.64 (64.8%)

## Data Availability

Data available on request from the authors.
